# Unique solvability of a crack problem with Signorini-type and Tresca friction conditions in a linearized elastodynamic body

**DOI:** 10.1098/rsta.2022.0225

**Published:** 2022-11-14

**Authors:** Takahito Kashiwabara, Hiromichi Itou

**Affiliations:** ^1^ Graduate School of Mathematical Sciences, The University of Tokyo, Tokyo 153-8914, Japan; ^2^ Department of Mathematics, Tokyo University of Science,Tokyo 162-8601, Japan

**Keywords:** dynamic linear elasticity, Signorini contact condition of dynamic type, Tresca friction condition

## Abstract

We consider dynamic motion of a linearized elastic body with a crack subject to a modified contact law, which we call *the Signorini contact condition of dynamic type*, and to the Tresca friction condition. Whereas the modified contact law involves both displacement and velocity, it formally includes the usual non-penetration condition as a special case. We prove that there exists a unique strong solution to this model. It is remarkable that not only existence but also uniqueness is obtained and that no viscosity term that serves as a parabolic regularization is added in our model.

This article is part of the theme issue ‘Non-smooth variational problems and applications’.

## 1. Introduction

Analysis of crack motion is one of the most important topics in fracture mechanics and it has also attracted much attention in material science or in seismology (e.g. [1–3]). However, at least from the mathematical point of view, it is far from being understood because of the highly nonlinear and singular behaviour of cracks. Even if we put aside problems regarding crack propagation, which are difficult even at the stage of modelling and will not be addressed in this paper, there still remain many mathematical difficulties as explained below.

In the static case, one of the basic models is the linearized elasticity with interfacial conditions representing the non-penetration contact law (also known as the Signorini condition) and the Coulomb friction law on the crack (see [4,5]). In principle, the former condition implies that normal stress acts on the crack only when its both sides are in contact, and the latter means that slip velocity across it occurs only when tangential stress reaches a threshold (see also remark 2.1 below). We leave two side remarks regarding this model: optimal regularity of weak solutions for the Signorini problem is obtained by Andersson [6] (see also [7]), and non-monotone friction laws that lead to hemi-variational inequalities can also be employed in place of the Coulomb law (see [8]).

The dynamical version of the above model, however, becomes much more difficult and no mathematical results seem to have been obtained. For related problems, in which some conditions mentioned above are modified or simplified, there are several known studies.

First, for the wave equation with the Signorini condition, unique solvability is established for the halfspace in [9]. Existence of a weak solution for general domains is proved by Kim [10], but uniqueness remains open. Generalization of these results to the linearized elasticity equations is also unsolved. If the Kelvin–Voigt viscoelastic model, in which a term serving as parabolic regularization is added to the linearized elasticity, is considered instead, then existence of a weak solution is obtained, e.g. in [11,12] and that of a strong solution is shown by Petrov & Schatzman [13]. If the contact law is furthermore modified in such a way that the Signorini condition is imposed on velocity rather than on displacement, then uniqueness of a weak solution is shown as well (see [11], Section 4.4.2).

Second, dynamic friction problems also exhibit a difficulty. In case of the Tresca friction law, where the threshold parameter of the tangential traction is a given function g, under the assumption that g does not depend on the time variable unique solvability of the linearized elasticity equations (without contact conditions) is established in [14]. This result was extended to the time-dependent g in our previous paper [15]. If the Coulomb friction law which is considered to be more realistic but is more complex is employed, in ([11], Chapter 5), existence of a solution to the Kelvin–Voigt viscoelastic model combined with the Signorini condition in velocity is proved. In the context of crack problems, a weak solution of the Kelvin–Voigt viscoelastic model with the Signorini condition in displacement and with the non-local (approximated) Coulomb friction law is constructed in [12,16].

Namely, when the contact condition is imposed on displacement and is combined with some friction law, only existence of a solution is established in the presence of viscosity terms. In view of such a situation, one would like to mathematically explore a dynamic elasticity model with contact and friction having the following properties:

(i) classical linear elasticity is exploited without viscosity;
(ii) not only existence but also uniqueness of a solution is ensured;
(iii) contact law is formulated in terms of displacement, which is considered to be more realistic.

In this paper, we propose to impose a contact condition to linear combination of normal displacement and normal velocity on the interface with some constant coefficient δ>0; see (2.2*a*) below. Since δ=0 and δ=∞ correspond to the contact conditions in displacement and in velocity, respectively, it can be regarded as an intermediate between them. We call (2.2*a*) *the Signorini contact condition of dynamic type* (hereinafter, referred to as *SCD condition*). With the SCD and Tresca friction conditions, we prove unique existence of a strong solution for the linearized elastodyanmic equations, thus having properties (i) and (ii). Moreover, property (iii) is also approached by our model because δ>0 can be fixed to an arbitrarily small value (however it is not possible to make exactly δ=0).

An expository interpretation of our result may be that making the Signorini contact condition in displacement ‘dynamic a bit’ (recall that boundary conditions having quantities with time derivative are called dynamic) leads to some stabilization effect to the system. We expect that this fact has some connection with Baumgarte-like stabilization techniques known in numerical simulations of non-smooth mechanics (see [17]), which is to be investigated in the future. The present result will also be of basic interest when we make an attempt to more involved crack problems, e.g. propagation and singular behaviour of crack tips.

This paper is organized as follows. In §2, we introduce notation and the precise mathematical setting to be studied. In §3, a variational inequality formulation as well as the definition of a strong solution is introduced, and we present the main theorem. Section 4 is devoted to its proof based on regularization of a variational inequality and Galerkin’s method. The strategy basically follows our previous study [15]; nevertheless, the analysis, in particular *a priori* estimates and a uniqueness proof, becomes more intricate to deal with the contact condition.

## 2. Preliminaries

### (a) Notation

Let $\Omega \subset R^{3}$ be a bounded domain with a smooth boundary ∂Ω consisting of two parts $\Gamma_{D}\neq \emptyset$ and $\Gamma_{N}$ that are mutually disjoint. Let Γ be a two-dimensional closed smooth interface that separates Ω into two subdomains $\Omega_{\pm }$, that is,

$$\Omega =\Omega_{+}\cup \Omega_{-}\cup \Gamma ,\;\Gamma ={\bar{\Omega }}_{+}\cap {\bar{\Omega }}_{-}.$$

**Figure 1.**
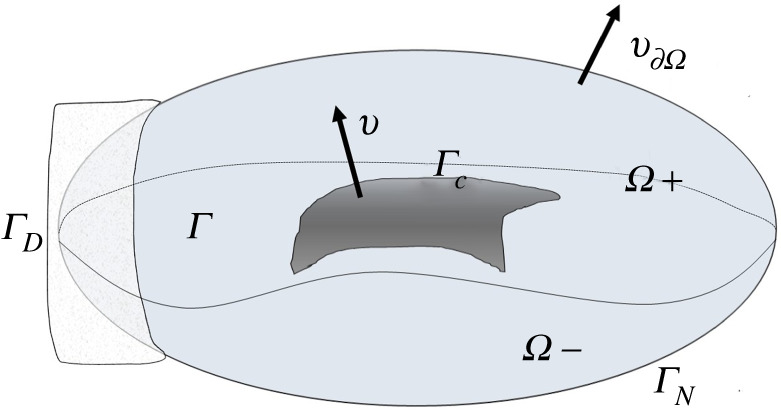
Domain with a crack.

We assume that $\partial \Omega_{\pm }$ satisfy the Lipschitz condition and that $\partial \Omega_{\pm }\cap \Gamma_{D}\neq \emptyset$. A crack is supposed to be represented by an open subset $\Gamma_{c}$ of Γ such that ${\bar{\Gamma }}_{c}\subset \Gamma \setminus \partial \Gamma$ (namely, $\Gamma_{c}⋐\Gamma$); we refer to $\Omega_{c}:=\Omega \setminus {\bar{\Gamma }}_{c}$ as *the domain with a crack*. The unit normal vector associated with ∂Ω is denoted by $\nu_{\partial \Omega }$, and the unit normal vector on Γ pointing from $\Omega_{-}$ to $\Omega_{+}$ is denoted by ν. The geometric situation explained so far is schematically summarized in figure 1.

We mainly deal with functions defined in $\Omega_{c}$ in this paper. For such a function u, we let $u^{\pm }:=u{|}_{\Omega_{\pm }}$ be its restrictions to subdomains $\Omega_{\pm }$. If $u^{\pm }$ are smooth enough, we define the jump discontinuity of u across Γ by

$$[[u]]:=u^{+}{|}_{\Gamma }-u^{-}{|}_{\Gamma },$$

and that of ∇u by $[[\nabla u]]:=(\nabla u^{+}){|}_{\Gamma }-(\nabla u^{-}){|}_{\Gamma }$.

For function spaces, we employ the usual Lebesgue spaces $L^{p}(\Omega_{c})\;(1\leq p\leq \infty )$ and the Sobolev space $H^{1}(\Omega_{c})$, which have the characterization

$$L^{p}(\Omega_{c})=L^{p}(\Omega_{+})\times L^{p}(\Omega_{-})$$

and

$$H^{1}(\Omega_{c})=\{(u^{+},u^{-})\in H^{1}(\Omega_{+})\times H^{1}(\Omega_{-}):[[u]]=0\text{ on }\Gamma \setminus \Gamma_{c}\}.$$

Accordingly, their norms are given by $\|u{\|}_{L^{p}(\Omega_{c})}:={\left(\|u^{+}{\|}_{L^{p}(\Omega_{+})}^{p}+\|u^{-}{\|}_{L^{p}(\Omega_{-})}^{p}\right)}^{1/p}$ and $\|u{\|}_{H^{1}(\Omega_{c})}:={\left(\|u^{+}{\|}_{H^{1}(\Omega_{+})}^{2}+\|u^{-}{\|}_{H^{1}(\Omega_{-})}^{2}\right)}^{1/2}$. Note in particular that if $u\in H^{1}(\Omega_{c})$ then $\left[[u]]\in H_{00}^{1/2}(\Gamma_{c}\right)$, which is the Lions–Magenes space (see [18]).

Functions and function spaces that are vector- or tensor-valued are written with bold fonts, e.g. $u\in H^{1}(\Omega_{c})=H^{1}{\left(\Omega_{c}\right)}^{3}$, whereas fine fonts mean scalar quantities. We denote the inner products of $L^{2}(\Omega_{c})$ by (⋅,⋅), and those of $L^{2}(\Gamma_{N}),L^{2}(\Gamma_{c})$ by ${\left(\cdot ,\cdot \right)}_{\Gamma_{N}},{\left(\cdot ,\cdot \right)}_{\Gamma_{c}}$ (the same notation will also be used for vectors and tensors). We also exploit the notation of Bochner spaces $L^{p}(0,T;X)$ and $W^{k,p}(0,T;X)$ for a positive constant T and a Banach space X, where k>0 is an integer and 1≤p≤∞. Finally, the dual space of X is denoted by $X^{*}$.

### (b) Problem formulation

We assume that $\Omega_{c}$ is regarded as a reference configuration (or non-deformed state) of an elastic body. The deformation of the body may be described by a displacement field $u:(0,T)\times \Omega_{c}\to R^{3}$. If the constitutive law of the material is based on isotropic linear elasticity, the stress tensor is given by

2.1 σ(u)=(λdiv⁡u)I+2μ E(u),

where λ,μ are Lamé constants such that μ>0 and 3λ+2μ>0, I is the unit tensor, and $E(u)=(\nabla u+{\left(\nabla u\right)}^{⊤})/2$ means the linearized strain tensor. The dynamic deformation of the body is governed by the hyperbolic system

$$\rho u^{\prime \prime }-\mathrm{div}\sigma (u)=\rho f\;\text{in}\;(0,T)\times \Omega_{c},$$

where ρ is the density which is a positive constant, the prime stands for the time derivative (i.e. $u^{\prime \prime }=\partial_{t}^{2}u$), f is the external body force and T>0 stands for a fixed time length. As for the boundary conditions, we consider

$$u=0\;\text{on}\;(0,T)\times \Gamma_{D}$$

and

$$\sigma (u)\nu_{\partial \Omega }=F\;\text{on}\;(0,T)\times \Gamma_{N},$$

where F is a prescribed traction on $\Gamma_{N}$. At t=0, the initial displacement and velocity fields are given as

$$u(0)=u_{0},\;u^{\prime }(0)={\dot{u}}_{0}\;\text{on}\;\{0\}\times \Omega_{c}.$$

Before stating the interface conditions on the crack, we introduce the normal and tangential components of the displacement, velocity and traction on Γ, restricted from $\Omega_{\pm }$, by

$$\begin{array}{rl} u_{\nu }^{\pm } & \;=u^{\pm }\cdot \nu ,\;u_{\tau }^{\pm }=u^{\pm }-u_{\nu }^{\pm }\nu ,\;u_{\nu }^{\prime \pm }=u^{\prime \pm }\cdot \nu ,\;u_{\tau }^{\prime \pm }=u^{\prime \pm }-u_{\nu }^{\prime \pm }\nu ,\\ \sigma_{\nu }^{\pm } & \;=\sigma (u^{\pm })\nu \cdot \nu ,\;\sigma_{\tau }^{\pm }=\sigma (u^{\pm })\nu -\sigma_{\nu }^{\pm }\nu , \end{array}$$

together with their jumps

$$\begin{array}{rl} & \;[[u_{\nu }]]=u_{\nu }^{+}-u_{\nu }^{-},\;[[u_{\tau }]]=u_{\tau }^{+}-u_{\tau }^{-},\;[[u_{\nu }^{\prime }]]=u_{\nu }^{\prime +}-u_{\nu }^{\prime -},\;[[u_{\tau }^{\prime }]]=u_{\tau }^{\prime +}-u_{\tau }^{\prime -},\\ & \;[[\sigma_{\nu }]]=[[\sigma_{\nu }(u)]]=\sigma_{\nu }^{+}-\sigma_{\nu }^{-},\;[[\sigma_{\tau }]]=[[\sigma_{\tau }(u)]]=\sigma_{\tau }^{+}-\sigma_{\tau }^{-}. \end{array}$$

In this paper, we consider the Signorini contact condition of dynamic type (SCD condition) and Tresca friction condition on the crack $\Gamma_{c}$ as follows:

2.2a $$[[\sigma_{\nu }]]=0,\;\sigma_{\nu }\leq 0,\;[[u_{\nu }+\delta u_{\nu }^{\prime }]]\geq 0,\;\sigma_{\nu }[[u_{\nu }+\delta u_{\nu }^{\prime }]]=0\;\text{on}\;(0,T)\times \Gamma_{c}$$

and

2.2b $$[[\sigma_{\tau }]]=0,\;|\sigma_{\tau }|\leq g,\;-\sigma_{\tau }\cdot [[u_{\tau }^{\prime }]]+g|[[u_{\tau }^{\prime }]]|=0\;\text{on}\;(0,T)\times \Gamma_{c},$$

where δ∈(0,∞] is a constant, g=g(t,x)≥0 is a given function.

Several remarks are in order. First, $\sigma_{\nu }:=\sigma_{\nu }^{+}=\sigma_{\nu }^{-}$ and $\sigma_{\tau }:=\sigma_{\tau }^{+}=\sigma_{\tau }^{-}$ are well-defined as single-valued functions on $\Gamma_{c}$ because they have no jump by (2.2). Second, if δ=0 in (2.2*a*) then we formally recover the usual non-penetration condition introduced in [5]. On the other hand, if δ=∞ then we arrive at the contact condition in terms of velocity given by Eck *et al.* [11]. To see this we equivalently rewrite (2.2*a*), with $\gamma :=\delta^{-1}$, as

2.3 $$\sigma_{\nu }\leq 0,\;[[\gamma u_{\nu }+u_{\nu }^{\prime }]]\geq 0,\;\sigma_{\nu }[[\gamma u_{\nu }+u_{\nu }^{\prime }]]=0,$$

and set γ=0. For simplicity of presentation, we mainly deal with the SCD condition in the form (2.3) with γ∈[0,∞) rather than (2.2*a*) in the subsequent analysis.

**Remark 2.1.**

(i) The introduction of δ in (2.2*a*) is mainly due to the mathematical reason as explained in the Introduction. From a modelling viewpoint, it can be regarded as a first-order approximation to the case δ=0, i.e. the usual non-penetration condition $[[u_{\nu }]]\geq 0$. We see that the SCD condition allows for interpenetration of the crack, which is not physically feasible and may be a restriction in applications. However, it remains realistic for a short time interval in the case of no initial slip velocity on the crack (e.g. for the first—and usually strongest—wave of an earthquake as mentioned in [11], Chapter 5).

(ii) If g in (2.2*b*) is replaced by $F|\sigma_{\nu }|$ (F≥0 is a coefficient), then the resulting condition is known as the *Coulomb friction law*, which is mentioned in the Introduction.

## 3. Variational formulations

### (a) Variational inequality

As discussed in the previous section, the strong form of the initial boundary value problem considered in this paper is represented as follows:

3.1a $$\begin{array}{rl} \rho u^{\prime \prime }-\mathrm{div}\sigma (u) & \;=\rho f\;\text{in}\;(0,T)\times \Omega_{c}, \end{array}$$

3.1b $$\begin{array}{rl} u & \;=0\;\text{on}\;(0,T)\times \Gamma_{D}, \end{array}$$

3.1c $$\begin{array}{rl} \sigma (u)\nu_{\partial \Omega } & \;=F\;\text{on}\;(0,T)\times \Gamma_{N}, \end{array}$$

3.1d $$\begin{array}{rl} \left[[\sigma_{\nu }]]=0,\;\sigma_{\nu }\leq 0,\;[[\gamma u_{\nu }+u_{\nu }^{\prime }]\right] & \;\geq 0,\;\sigma_{\nu }[[\gamma u_{\nu }+u_{\nu }^{\prime }]]=0\;\text{on}\;(0,T)\times \Gamma_{c}, \end{array}$$

3.1e $$\begin{array}{rl} {}[[\sigma_{\tau }]]=0,\;|\sigma_{\tau }| & \;\leq g,\;\sigma_{\tau }\cdot [[u_{\tau }^{\prime }]]=g|[[u_{\tau }^{\prime }]]|\;\text{on}\;(0,T)\times \Gamma_{c}, \end{array}$$

3.1f $$\begin{array}{rl} u(0)=u_{0},\;u^{\prime }(0) & \;={\dot{u}}_{0}\;\text{on}\;\{0\}\times \Omega_{c}. \end{array}$$

Let us derive a weak formulation to this problem assuming that u is smooth enough in $\left[0,T]\times (\bar{\Omega }\setminus \Gamma_{c}\right)$. To this end we introduce the following function spaces and convex cone:

$$H:=L^{2}(\Omega_{c}),\;V:=\{v\in H^{1}(\Omega_{c}):v=0\text{ on }\Gamma_{D}\},\;K:=\{v\in V:[[v_{\nu }]]\geq 0\text{ a.e.\textbackslash{} on }\Gamma_{c}\}.$$

Multiplying (3.1*a*) by $v-(\gamma u+u^{\prime })$ with an arbitrary v∈K and integrating over $\Omega_{c}$, we obtain

$$\begin{array}{rl} & \rho (u^{\prime \prime }(t),v-(\gamma u(t)+u^{\prime }(t)))+(\sigma (u(t)),\nabla (v-(\gamma u(t)+u^{\prime }(t))))\\ & \;\;+{(\sigma_{\nu }(t),[[v_{\nu }-(\gamma u_{\nu }(t)+u_{\nu }^{\prime }(t))]])}_{\Gamma_{c}}+{(\sigma_{\tau }(t),[[v_{\tau }-(\gamma u_{\tau }(t)+u_{\tau }^{\prime }(t))]])}_{\Gamma_{c}}\\ & \;=\rho (f(t),v-(\gamma u(t)+u^{\prime }(t)))+{(F(t),v-(\gamma u(t)+u^{\prime }(t)))}_{\Gamma_{N}}\;\forall t\in (0,T), \end{array}$$

where we have used $[[\sigma_{\nu }]]=0,[[\sigma_{\tau }]]=0$ on $\Gamma_{c}$ and the fact that the outer unit normal w.r.t. $\Omega_{\pm }$ on Γ is ∓ν. By (2.1) we see that

(σ(u),∇v)=(σ(u),E(v))=λ(div⁡u,div⁡v)+2μ(E(u),E(v))=:a(u,v)∀v∈V.

It follows from (3.1*d*) and (3.1*e*) that

3.2 $${(\sigma_{\nu }(u(t)),[[v_{\nu }-(\gamma u_{\nu }(t)+u_{\nu }^{\prime }(t))]])}_{\Gamma_{c}}\leq 0$$

and

3.3 $${(\sigma_{\tau }(u(t)),[[v_{\tau }-(\gamma u_{\tau }(t)+u_{\tau }^{\prime }(t))]])}_{\Gamma_{c}}\leq {(g(t),|[[v_{\tau }-\gamma u_{\tau }(t)]]|-|[[u_{\tau }^{\prime }(t)]]|)}_{\Gamma_{c}}.$$

Consequently,

3.4 $$\begin{array}{rl} & \rho (u^{\prime \prime }(t),v-(\gamma u(t)+u^{\prime }(t)))+a(u(t),v-(\gamma u(t)+u^{\prime }(t)))\\ & \;\;+{(g(t),|[[v_{\tau }-\gamma u_{\tau }(t)]]|-|[[u_{\tau }^{\prime }(t)]]|)}_{\Gamma_{c}}\\ & \;\geq \rho (f(t),v-(\gamma u(t)+u^{\prime }(t)))+{(F(t),v-(\gamma u(t)+u^{\prime }(t)))}_{\Gamma_{N}}\;\forall v\in V,\;\text{a.e.}\;t\in (0,T). \end{array}$$

This is a variational inequality of hyperbolic type that is equivalent to the strong form (3.1), provided that there is a classical solution, as seen below.

**Proposition 3.1.**

*Let* u *be smooth enough to satisfy* $u\in C^{2}([0,T]\times (\bar{\Omega }\setminus \Gamma_{c}))$. *Then* u *solves (3.1) if and only if the following hold*:

(i) u(t)∈V *for all* t∈(0,T);
(ii) $u(0)=u_{0}$ *and* $u^{\prime }(0)={\dot{u}}_{0}$;
(iii) $\gamma u(t)+u^{\prime }(t)\in K$ *for all* t∈(0,T);
(iv) u *satisfies the hyperbolic variational inequality* (3.4).

**Proof.**

The proof is essentially similar to ([15, pp. 125–126]). It suffices to show the ‘if’ part. Taking a test function $v=\pm w+\gamma u(t)+u^{\prime }(t)$ with arbitrary w∈V such that [[w]]=0 on Γ, one can reduce (3.4) to

$$\rho (u^{\prime \prime }(t),w)+a(u(t),w)=\rho (f(t),w)+{\left(F(t),w\right)}_{\Gamma_{N}},$$

which implies (3.1*a*), (3.1*c*), $[[\sigma_{\nu }]]=0$ on $\Gamma_{c}$, and $[[\sigma_{\tau }]]=0$ on $\Gamma_{c}$. Then (3.2) and (3.3) follow from integration by parts (note that each of $\left[[v_{\nu }]\right]$ and $\left[[v_{\tau }]\right]$ can be chosen to an arbitrary smooth function independently).

First we focus on (3.2). Setting $\left[[v_{\nu }]\right]$ to 0 and $2[[\gamma u_{\nu }(t)+u_{\nu }^{\prime }(t)]]$ gives

$${\left(\sigma_{\nu }(t),[[\gamma u_{\nu }(t)+u_{\nu }^{\prime }(t)]]\right)}_{\Gamma_{c}}=0.$$

Therefore, $(\sigma_{\nu }{\left(t),[[v_{\nu }]]\right)}_{\Gamma_{c}}\leq 0$ for arbitrary $[[v_{\nu }]]\geq 0$, which implies

$$\sigma_{\nu }(t)\leq 0\;\text{on}\;\Gamma_{c}.$$

These two relations combined with $[[\gamma u_{\nu }(t)+u_{\nu }^{\prime }(t)]]\geq 0$ on $\Gamma_{c}$ deduce the last equality of (3.1*d*).

Next, in (3.3), setting $\left[[v_{\tau }]\right]$ to $\left[[\gamma u_{\tau }(t)]\right]$ and $\left[[\gamma u_{\tau }(t)+2u_{\tau }^{\prime }(t)]\right]$ gives

$${(\sigma_{\tau }(t),[[u_{\tau }^{\prime }(t)]])}_{\Gamma_{c}}={(g(t),|[[u_{\tau }^{\prime }(t)]]|)}_{\Gamma_{c}}.$$

Therefore, ${\left(\sigma_{\tau }(t),[[v_{\tau }]]\right)}_{\Gamma_{c}}\leq {\left(g(t),|[[v_{\tau }]]|\right)}_{\Gamma_{c}}$ for arbitrary $\left[[v_{\tau }]\right]$, which implies $|\sigma_{\tau }(t)|\leq g(t)$ on $\Gamma_{c}$. Then the last equality of (3.1*e*) also follows. This proves that u solves (3.1).

### (b) Main result

In view of proposition 3.1, let us define a solution of (3.1) based on its variational form.

**Definition 3.2.**

Given f, F, g, $u_{0}$, ${\dot{u}}_{0}$, we say that $u\in W^{2,\infty }(0,T;H)\cap W^{1,\infty }(0,T;V)$ is a strong solution of (3.1) if u satisfies conditions (i)–(iv) in proposition 3.1.

**Remark 3.3.**

For second-order hyperbolic problems, one usually considers a weak solution in $W^{1,\infty }(0,T;L^{2}(\Omega_{c}))\cap L^{\infty }(0,T;H^{1}(\Omega_{c}))$. However, this class would not be appropriate for dynamic elasticity problems with friction where the trace of velocity explicitly appears on an interface. We also note that in the Kelvin–Voigt viscoelastic case, a natural class of a weak solution becomes $W^{1,\infty }(0,T;L^{2}(\Omega_{c}))\cap H^{1}(0,T;H^{1}(\Omega_{c}))$, avoiding this issue.

Now we are ready to state our main result in this paper.

**Theorem 3.4.**

*Let* γ∈[0,∞), $f\in H^{1}(0,T;H),F\in H^{2}(0,T;L^{2}(\Gamma_{N}))$, *and let* $g\in H^{2}(0,T;L^{2}(\Gamma_{c}))$ *be non-negative. We assume that* $u_{0}\in V$, ${\dot{u}}_{0}\in V$ *and that they satisfy the following compatibility conditions*:

— $-\mathrm{div}\sigma (u_{0})\in H$;
— $\sigma (u_{0})\nu_{\partial \Omega }=F(0)$ *on* $\Gamma_{N}$;
— $\sigma_{\nu }(u_{0}^{+})=\sigma_{\nu }(u_{0}^{-})=0$ *and* $[[\gamma u_{0\nu }+{\dot{u}}_{0\nu }]]=0$ on $\Gamma_{c}$;
— $\sigma_{\tau }(u_{0}^{+})=\sigma_{\tau }(u_{0}^{-})=0$ *and* $[[{\dot{u}}_{0\tau }]]=0$ *on* $\Gamma_{c}$.

*Then there exists a unique strong solution of (3.1)*.

**Remark 3.5.**

Since $u_{0}\in V$ satisfies $-\mathrm{div}\sigma (u_{0}^{\pm })\in L^{2}(\Omega_{\pm })$, initial tractions $\sigma (u_{0}^{\pm })\nu_{\partial \Omega }$ and $\sigma (u_{0}^{\pm })\nu$ are well-defined in ${\left(H_{00}^{1/2}(\Gamma_{N})\right)}^{*}$ and ${\left(H_{00}^{1/2}(\Gamma_{c})\right)}^{*}$, respectively. The third and fourth conditions above are stronger than just requiring that $u_{0}$ and ${\dot{u}}_{0}$ satisfy (3.1*d*) and (3.1*e*) at t=0; however, we are not aware whether they can be weakened.

### (c) Regularized problem

It is not easy to directly construct a solution of the time-dependent variational inequality (3.4) because it contains non-differentiable relations. To see this, we introduce two convex functions

$$\psi (x)=\left\{ \begin{array}{ll} +\infty & (x<0),\\ 0 & (x\geq 0), \end{array}\right.\;\varphi (x)=|x|\;(x\in R^{3}),$$

whose subdifferentials β:=∂ψ and α:=∂φ are maximal monotone graphs given by

$$\beta (x)=\left\{ \begin{array}{ll} \emptyset & x<0,\\ \left(-\infty ,0\right] & x=0,\\ 0 & x>0, \end{array}\right.\;\alpha (x)=\left\{ \begin{array}{ll} x/|x| & (x\neq 0),\\ \left\{y\in R^{3}:|y|\leq 1\right\} & (x=0). \end{array}\right.$$

We then observe that the SCD and Tresca conditions in (3.1) are concisely expressed as

3.5 $$\sigma_{\nu }\in \beta ([[\gamma u_{\nu }+u_{\nu }^{\prime }]]),\;\sigma_{\tau }\in g\alpha ([[u_{\tau }^{\prime }]]).$$

To address the difficulty that β and α are multi-valued functions and non-differentiable, we approximate ψ and φ by the following functions which are convex and $W^{3,\infty }\cap C^{2}$:

$$\psi_{\epsilon }(x)=\frac{1}{3\epsilon }{\left[x\right]}_{-}^{3},\;\varphi_{\epsilon }(x)=\sqrt{|x{|}^{2}+\epsilon^{2}},$$

where ϵ>0 is a constant and ${\left[x\right]}_{-}:=\max\{-x,0\}$ for x∈R. Their derivatives $\beta_{\epsilon }:=d\psi_{\epsilon }/dx$ and $\alpha_{\epsilon }:=\nabla \varphi_{\epsilon }$ are given by

$$\beta_{\epsilon }(x)=-\frac{1}{\epsilon }{\left[x\right]}_{-}^{2},\;\alpha_{\epsilon }(x)=\frac{x}{\sqrt{|x{|}^{2}+\epsilon^{2}}},$$

which are monotone and $W^{2,\infty }\cap C^{1}$.

With this preparation we consider the following regularized problem denoted by **(VI)${\;}_{\epsilon }$**: find $u_{\epsilon }(t)\in V$ such that $u_{\epsilon }(0)=u_{0},u_{\epsilon }^{\prime }(0)={\dot{u}}_{0}$ and

3.6 $$\begin{array}{rl} & \rho (u_{\epsilon }^{\prime \prime }(t),v-(\gamma u_{\epsilon }(t)+u_{\epsilon }^{\prime }(t)))+a(u_{\epsilon }(t),v-(\gamma u_{\epsilon }(t)+u_{\epsilon }^{\prime }(t)))\\ & \;\;+{(1,\psi_{\epsilon }([[v_{\nu }]])-\psi_{\epsilon }([[\gamma u_{\epsilon \nu }(t)+u_{\epsilon \nu }^{\prime }(t)]]))}_{\Gamma_{c}}+{(g(t),\varphi_{\epsilon }([[v_{\tau }-\gamma u_{\epsilon \tau }(t)]])-\varphi_{\epsilon }([[u_{\epsilon \tau }^{\prime }(t)]]))}_{\Gamma_{c}}\\ & \;\geq \rho (f(t),v-(\gamma u_{\epsilon }(t)+u_{\epsilon }^{\prime }(t)))+(F(t),v-{\left(\gamma u_{\epsilon }(t)+u_{\epsilon }^{\prime }(t))\right)}_{\Gamma_{N}}\;\forall v\in V,\;\text{a.e.}\;t\in (0,T). \end{array}$$

In the proposition below, we find that ${\text{( VI) }}_{\epsilon }$ is equivalent to the following variational equality problem denoted by ${\text{(VE)}}_{\epsilon }$: find $u_{\epsilon }(t)\in V$ such that $u_{\epsilon }(0)=u_{0},u_{\epsilon }^{\prime }(0)={\dot{u}}_{0}$ and

3.7 $$\begin{array}{rl} & \rho (u_{\epsilon }^{\prime \prime }(t),v)+a(u_{\epsilon }(t),v)+{(\beta_{\epsilon }([[\gamma u_{\epsilon \nu }(t)+u_{\epsilon \nu }^{\prime }(t)]]),[[v_{\nu }]])}_{\Gamma_{c}}+{(g(t)\alpha_{\epsilon }([[u_{\epsilon \tau }^{\prime }(t)]]),[[v_{\tau }]])}_{\Gamma_{c}}\\ & \;=\rho (f{\left(t),v)+(F(t),v\right)}_{\Gamma_{N}}\;\forall v\in V,\;\text{a.e.}\;t\in (0,T). \end{array}$$

**Proposition 3.6.**

*Let* $u_{\epsilon }\in W^{2,\infty }(0,T;H)\cap W^{1,\infty }(0,T;V)$. *It solves* ${\text{(VI)}}_{\epsilon }$ *if and only if it solves* ${\text{( VE) }}_{\epsilon }$.

**Proof.**

Although the proof is standard, we present it for completeness. Let $u_{\epsilon }$ be a solution of ${\text{(VI)}}_{\epsilon }$. Taking $v=\pm hw+\gamma u_{\epsilon }(t)+u_{\epsilon }^{\prime }(t)$ with arbitrary h>0 and w∈V, dividing by h, and letting h→0, we deduce ${\text{(VE)}}_{\epsilon }$ from the relations

$$\lim_{h\to 0}\frac{\psi_{\epsilon }([[hw_{\nu }+\gamma u_{\epsilon \nu }(t)+u_{\epsilon \nu }^{\prime }(t)]])-\psi_{\epsilon }([[\gamma u_{\epsilon \nu }(t)+u_{\epsilon \nu }^{\prime }(t)]])}{h}=\beta_{\epsilon }([[\gamma u_{\epsilon \nu }(t)+u_{\epsilon \nu }^{\prime }(t)]])\;[[w_{\nu }]]$$

and

$$\lim_{h\to 0}\frac{\varphi_{\epsilon }([[hw_{\tau }+u_{\epsilon \tau }^{\prime }(t)]])-\varphi_{\epsilon }([[u_{\epsilon \tau }^{\prime }(t)]])}{h}=\alpha_{\epsilon }([[u_{\epsilon \tau }^{\prime }(t)]])\cdot [[w_{\tau }]].$$

Conversely, let $u_{\epsilon }$ be a solution of **(VE)${\;}_{\epsilon }$**. Note that, since $\psi_{\epsilon }$ and $\varphi_{\epsilon }$ are convex,

$$\psi_{\epsilon }([[w_{\nu }+\gamma u_{\epsilon \nu }(t)+u_{\epsilon \nu }^{\prime }(t)]])-\psi_{\epsilon }([[\gamma u_{\epsilon \nu }(t)+u_{\epsilon \nu }^{\prime }(t)]])\geq \beta_{\epsilon }([[\gamma u_{\epsilon \nu }(t)+u_{\epsilon \nu }^{\prime }(t)]])\;[[w_{\nu }]]$$

and

$$\varphi_{\epsilon }([[w_{\tau }+u_{\epsilon \tau }^{\prime }(t)]])-\varphi_{\epsilon }([[u_{\epsilon \tau }^{\prime }(t)]])\geq \alpha_{\epsilon }([[u_{\epsilon \tau }^{\prime }(t)]])\cdot [[w_{\tau }]],$$

for all w∈V. Setting this w in such a way that $w+\gamma u_{\epsilon }(t)+u_{\epsilon }^{\prime }(t)=v$ and using (3.7), we arrive at (3.6).

As a result of proposition 3.6, it suffices to solve an equation problem for obtaining $u_{\epsilon }$. Furthermore, since it follows from (3.7) that

$$\sigma_{\nu }(u_{\epsilon })=\beta_{\epsilon }([[\gamma u_{\epsilon \nu }+u_{\epsilon \nu }^{\prime }]]),\;\sigma_{\tau }(u_{\epsilon })=\alpha_{\epsilon }([[u_{\epsilon \tau }^{\prime }]])\;\text{on}\;(0,T)\times \Gamma_{c},$$

we expect that $u_{\epsilon }$ should converge to a solution of the original problem (3.1) as ϵ→0. Justification of this fact, which is actually the idea to prove theorem 3.4, is the task of the next section.

## 4. Proof of main result

In this section, we establish existence in §4a–d and uniqueness in §4e. Coercivity of a(⋅,⋅) in V, that is,

$$a(v,v)\geq C\|v{\|}_{{\text{H}}^{\text{1}}(\Omega_{c})}^{2}\;\forall v\in V,$$

which is justified by Korn’s inequality (e.g. [14]), will be frequently used in the proof. Here and in what follows, C represents a generic constant depending only on the domain $\Omega_{c}$, Lamé constants λ, μ and density ρ. We will also write C(f,g), etc. in order to indicate dependency on other quantities.

The inequality above allows us to define the norm of V as $\|v{\|}_{V}:=a{\left(v,v\right)}^{1/2}$, whereas we use $\|v{\|}_{H}:=\|v{\|}_{L^{2}(\Omega_{c})}$.

### (a) Galerkin approximation

We apply Galerkin’s method to solve (3.7). Since $V\subset H^{1}(\Omega_{c})$ is separable, there exist countable members $w_{1},w_{2},\ldots ,\in V$, which are linearly independent, such that $\bar{\overset{\infty }{\underset{m=1}{⋃}}V_{m}}=V$ where $V_{m}:=\mathrm{span}{\left\{w_{k}\right\}}_{k=1}^{m}$. We may assume that $u_{0},{\dot{u}}_{0}\in V_{m}$ for m≥2 (otherwise one can add $u_{0}$ and ${\dot{u}}_{0}$ to the members ${\left\{w_{k}\right\}}_{k=1}^{m}$).

For m=2,3,…, the Galerkin approximation problem consists in determining $c_{k}(t)\;(k=1,\ldots ,m)$ such that $u_{m}=\sum_{k=1}^{m}c_{k}(t)w_{k}(x)\in V_{m}$ satisfies

4.1 $$\begin{array}{rl} & \rho (u_{m}^{\prime \prime }(t),v)+a(u_{m}(t),v)+{(\beta_{\epsilon }([[\gamma u_{m\nu }(t)+u_{m\nu }^{\prime }(t)]]),[[v_{\nu }]])}_{\Gamma_{c}}+{(g(t)\alpha_{\epsilon }([[u_{m\tau }^{\prime }(t)]]),[[v_{\tau }]])}_{\Gamma_{c}}\\ & \;=\rho (f(t),v)+{\left(F(t),v\right)}_{\Gamma_{N}}\;\forall v\in V_{m},\;\forall t\in (0,T), \end{array}$$

together with the initial conditions $u_{m}(0)=u_{0},u_{m}^{\prime }(0)={\dot{u}}_{0}$.

This is a finite-dimensional system of ODEs that admits a local-in-time unique solution

$$c_{k}\in W^{3,\infty }(0,\tilde{T})\cap C^{2}([0,\tilde{T}])\;(k=1,\ldots ,m),$$

for certain $0<\tilde{T}\leq T$ (recall that $\beta_{\epsilon }$, $\alpha_{\epsilon }$ are $W^{2,\infty }\cap C^{1}$). Because the *a priori* estimates below ensure that $\tilde{T}$ can be extended to T, we use T instead of $\tilde{T}$ from the beginning.

Differentiating (4.1) in t we obtain

4.2 $$\begin{array}{rl} & \rho (u_{m}^{\prime \prime \prime }(t),v)+a(u_{m}^{\prime }(t),v)+{(\beta_{\epsilon }^{\prime }([[\gamma u_{m\nu }(t)+u_{m\nu }^{\prime }(t)]])[[\gamma u_{m\nu }^{\prime }(t)+u_{m\nu }^{\prime \prime }(t)]],[[v_{\nu }]])}_{\Gamma_{c}}\\ & \;\;+{(g^{\prime }(t)\alpha_{\epsilon }([[u_{m\tau }^{\prime }(t)]]),[[v_{\tau }]])}_{\Gamma_{c}}+{(g(t)\nabla \alpha_{\epsilon }([[u_{m\tau }^{\prime }(t)]])[[u_{m\tau }^{\prime \prime }(t)]],[[v_{\tau }]])}_{\Gamma_{c}}\\ & \;=\rho (f^{\prime }(t),v)+{\left(F^{\prime }(t),v\right)}_{\Gamma_{N}}\;\forall v\in V_{m},\;\forall t\in (0,T). \end{array}$$

### (b) First *a priori* estimate

Let us establish an estimate for $u_{m}\in W^{1,\infty }(0,T;H)\cap L^{\infty }(0,T;V)$. For arbitrary t∈(0,T) take $v=\gamma u_{m}+u_{m}^{\prime }\in V_{m}$ in (4.1) to obtain

$$\begin{array}{rl} & \frac{1}{2}\frac{d}{dt}(\rho \|u_{m}^{\prime }(t){\|}_{H}^{2}+\|u_{m}(t){\|}_{V}^{2})+\gamma \|u_{m}(t){\|}_{V}^{2}+\frac{1}{\epsilon }\|{\left[[\gamma u_{m\nu }(t)+u_{m\nu }^{\prime }(t)]\right]}_{-}{\|}_{L^{3}(\Gamma_{c})}^{3}\\ & \;\;+\rho \gamma (u_{m}^{\prime \prime }(t),u_{m}(t))\leq \rho (f(t),\gamma u_{m}(t)+u_{m}^{\prime }(t))+{\left(F(t),\gamma u_{m}(t)+u_{m}^{\prime }(t)\right)}_{\Gamma_{N}}\\ & \;\;-\gamma {(g(t)\alpha_{\epsilon }([[u_{m\tau }^{\prime }(t)]]),[[u_{m\tau }(t)]])}_{\Gamma_{c}}, \end{array}$$

where ${\left[[v]\right]}_{-}$ means ${\left[[[v]]\right]}_{-}$ and we have used $\beta_{\epsilon }(x)x=\frac{1}{\epsilon }{\left[x\right]}_{-}^{3},\alpha_{\epsilon }(x)\cdot x\geq 0$. Applying Hölder’s and Young’s inequalities to terms involving γ on the right-hand side yields

$$\begin{array}{rl} & \frac{1}{2}\frac{d}{dt}(\rho \|u_{m}^{\prime }(t){\|}_{H}^{2}+\|u_{m}(t){\|}_{V}^{2})+\frac{\gamma }{2}\|u_{m}(t){\|}_{V}^{2}+\frac{1}{\epsilon }\|{\left[[\gamma u_{m\nu }(t)+u_{m\nu }^{\prime }(t)]\right]}_{-}{\|}_{L^{3}(\Gamma_{c})}^{3}\\ & \;\;+\rho \gamma (u_{m}^{\prime \prime }(t),u_{m}(t))\leq C\gamma (\|f(t){\|}_{H}^{2}+\|F(t){\|}_{L^{2}(\Gamma_{N})}^{2}+\|g(t){\|}_{L^{2}(\Gamma_{c})}^{2})\\ & \;\;+\rho (f(t),u_{m}^{\prime }(t))+{\left(F(t),u_{m}^{\prime }(t)\right)}_{\Gamma_{N}}, \end{array}$$

where we have used $|\alpha_{\epsilon }(\cdot )|\leq 1$ and the trace inequality $\|[[v]]{\|}_{L^{2}(\Gamma_{c})}\leq C\|v{\|}_{V}$. Integration of both sides with respect to t gives

$$\begin{array}{rl} & \frac{1}{2}(\rho \|u_{m}^{\prime }(t){\|}_{H}^{2}+\|u_{m}(t){\|}_{V}^{2})+\frac{\gamma }{2}\int_{0}^{t}\|u_{m}(s){\|}_{V}^{2}\;ds\\ & \;\;+\frac{1}{\epsilon }\int_{0}^{t}\|{\left[[\gamma u_{m\nu }(s)+u_{m\nu }^{\prime }(s)]\right]}_{-}{\|}_{L^{3}(\Gamma_{c})}^{3}\;ds\\ & \;\;+\rho \gamma {\left[(u_{m}^{\prime }(s),u_{m}(s))\right]}_{0}^{t}-\rho \gamma \int_{0}^{t}\|u_{m}^{\prime }(s){\|}_{H}^{2}\;ds\\ & \;\leq \frac{1}{2}(\rho \|{\dot{u}}_{0}{\|}_{H}^{2}+\|u_{0}){\|}_{V}^{2})+C\gamma (\|f{\|}_{L^{2}(0,T;H)}^{2}+\|F{\|}_{L^{2}(0,T;L^{2}(\Gamma_{N}))}^{2}+\|g{\|}_{L^{2}(0,T;L^{2}(\Gamma_{c}))}^{2})\\ & \;\;+\frac{\rho }{2}\|f{\|}_{L^{2}(0,T;H)}^{2}+\frac{\rho }{2}\int_{0}^{t}\|u_{m}^{\prime }(s){\|}_{H}^{2}\;ds+{\left[{\left(F(s),u_{m}(s)\right)}_{\Gamma_{N}}\right]}_{0}^{t}-\int_{0}^{t}{\left(F^{\prime }(s),u_{m}(s)\right)}_{\Gamma_{N}}\;ds. \end{array}$$

In particular,

$$\begin{array}{rl} & \rho \|u_{m}^{\prime }(t){\|}_{H}^{2}+\frac{1}{2}\|u_{m}(t){\|}_{V}^{2}+\rho \gamma \frac{d}{dt}\|u_{m}(t){\|}_{H}^{2}+\frac{2}{\epsilon }\int_{0}^{t}\|{\left[[\gamma u_{m\nu }(s)+u_{m\nu }^{\prime }(s)]\right]}_{-}{\|}_{L^{3}(\Gamma_{c})}^{3}\;ds\\ & \;\leq C(\gamma +1)(\|f{\|}_{L^{2}(0,T;H)}^{2}+\|F{\|}_{H^{1}(0,T;L^{2}(\Gamma_{N}))}^{2}+\|g{\|}_{L^{2}(0,T;L^{2}(\Gamma_{c}))}^{2}+\|u_{0}{\|}_{V}^{2}+\|{\dot{u}}_{0}{\|}_{H}^{2})\\ & \;\;+C(\gamma +1)\int_{0}^{t}(\rho \|u_{m}^{\prime }(s){\|}_{H}^{2}+\frac{1}{2}\|u_{m}(s){\|}_{V}^{2})\;ds, \end{array}$$

where ${\left(F(t),u_{m}(t)\right)}_{\Gamma_{N}}$ has been bounded by $C\|F{\|}_{H^{1}(0,T;L^{2}(\Gamma_{N}))}^{2}+\frac{1}{4}\|u_{m}(t){\|}_{V}^{2}$. Setting $A(t):=\rho \|u_{m}^{\prime }(t){\|}_{H}^{2}+\frac{1}{2}\|u_{m}(t){\|}_{V}^{2}$ and neglecting the last term on the left-hand side (this is just for simplicity of presentation; if we keep this term, we obtain (4.5) below), we rephrase this estimate as

4.3 $$A(t)+\rho \gamma \frac{d}{dt}\|u_{m}(t){\|}_{H}^{2}\leq C_{1}(f,F,g,u_{0},{\dot{u}}_{0})(\gamma +1)+C(\gamma +1)\int_{0}^{t}A(s)\;ds\;\forall t\in (0,T).$$

If γ=0, we find from Gronwall’s inequality that

$$A(t)\leq C_{1}(f,F,g,u_{0},{\dot{u}}_{0})\;e^{Ct}.$$

Otherwise we further integrate (4.3) with respect to t, with $B_{1}(t):=\int_{0}^{t}A(s)\;ds$, to get

$$B_{1}(t)+\rho \gamma \|u_{m}(t){\|}_{H}^{2}\leq C_{2}(f,F,g,u_{0},{\dot{u}}_{0},T)(\gamma +1)+C(\gamma +1)\int_{0}^{t}B_{1}(s)\;ds,$$

so that, by Gronwall’s inequality,

$$B_{1}(t)+\rho \gamma \|u_{m}(t){\|}_{H}^{2}\leq C_{2}(f,F,g,u_{0},{\dot{u}}_{0},T)(\gamma +1)\;e^{C(\gamma +1)t}.$$

Since $\rho \gamma (d/dt)\|u_{m}(t){\|}_{H}^{2}=2\rho \gamma (u_{m}^{\prime }(t),u_{m}(t))$, we find from (4.3) that

$$A(t)\leq C_{1}(f,F,g,u_{0},{\dot{u}}_{0})(\gamma +1)+C(\gamma +1)B_{1}(t)+\frac{\rho }{2}\|u_{m}^{\prime }(t){\|}_{H}^{2}+2\rho \gamma^{2}\|u_{m}(t){\|}_{H}^{2},$$

which concludes

4.4 $$\frac{1}{2}(\rho \|u_{m}^{\prime }(t){\|}_{H}^{2}+\|u_{m}(t){\|}_{V}^{2})\leq C_{3}(f,F,g,u_{0},{\dot{u}}_{0},T){\left(\gamma +1\right)}^{2}\;e^{C(\gamma +1)t}.$$

**Remark 4.1.**

As we already noted before (4.3), it also holds that, for all t∈[0,T],

4.5 $$\frac{2}{\epsilon }\int_{0}^{t}\|{\left[[\gamma u_{m\nu }(s)+u_{m\nu }^{\prime }(s)]\right]}_{-}{\|}_{L^{3}(\Gamma_{c})}^{3}\;ds\leq C_{3}(f,F,g,u_{0},{\dot{u}}_{0},T){\left(\gamma +1\right)}^{2}\;e^{C(\gamma +1)t}.$$

### (c) Second *a priori* estimate

Next let us establish an estimate for $u_{m}^{\prime }\in W^{1,\infty }(0,T;H)\cap L^{\infty }(0,T;V)$. For arbitrary t∈(0,T) we take $v=\gamma u_{m}^{\prime }+u_{m}^{\prime \prime }\in V_{m}$ in (4.2) to obtain

$$\begin{array}{rl} & \frac{1}{2}\frac{d}{dt}(\rho \|u_{m}^{\prime \prime }(t){\|}_{H}^{2}+\|u_{m}^{\prime }(t){\|}_{V}^{2})+\gamma \|u_{m}^{\prime }(t){\|}_{V}^{2}+\gamma \rho (u_{m}^{\prime \prime \prime }(t),u_{m}^{\prime }(t))\\ & \;\;\leq \gamma \rho (f^{\prime }(t),u_{m}^{\prime }(t))+\gamma {\left(F^{\prime }(t),u_{m}^{\prime }(t)\right)}_{\Gamma_{N}}+\rho (f^{\prime }(t),u_{m}^{\prime \prime }(t))+{\left(F^{\prime }(t),u_{m}^{\prime \prime }(t)\right)}_{\Gamma_{N}}\\ & \;\;-\gamma {(g(t)\nabla \alpha_{\epsilon }([[u_{m\tau }^{\prime }(t)]])[[u_{m\tau }^{\prime \prime }(t)]],[[u_{m\tau }^{\prime }(t)]])}_{\Gamma_{c}}-\gamma {(g^{\prime }(t)\alpha_{\epsilon }([[u_{m\tau }^{\prime }(t)]]),[[u_{m\tau }^{\prime }]])}_{\Gamma_{c}}\\ & \;\;-{(g^{\prime }(t)\alpha_{\epsilon }([[u_{m\tau }^{\prime }(t)]]),[[u_{m\tau }^{\prime \prime }]])}_{\Gamma_{c}}, \end{array}$$

where we have used the fact that $\beta_{\epsilon }^{\prime }$ and $\nabla \alpha_{\epsilon }$ are non-negative.

Applying Hölder’s and Young’s inequalities to the first three and the sixth terms on the right-hand side, together with $|\alpha_{\epsilon }(\cdot )|\leq 1$ and the trace inequality $\|[[v]]{\|}_{L^{2}(\Gamma_{c})}\leq C\|v{\|}_{V}$, we have

$$\begin{array}{rl} & \frac{1}{2}\frac{d}{dt}(\rho \|u_{m}^{\prime \prime }(t){\|}_{H}^{2}+\|u_{m}^{\prime }(t){\|}_{V}^{2})+\frac{\gamma }{2}\|u_{m}^{\prime }(t){\|}_{V}^{2}+\rho \gamma (u_{m}^{\prime \prime \prime }(t),u_{m}^{\prime }(t))\\ & \;\;\leq C(\gamma +1)(\|f^{\prime }(t){\|}_{H}^{2}+\|F^{\prime }(t){\|}_{L^{2}(\Gamma_{N})}^{2}+\|g^{\prime }(t){\|}_{L^{2}(\Gamma_{c})}^{2})+\rho \|u_{m}^{\prime \prime }(t){\|}_{H}^{2}\\ & \;\;+{\left(F^{\prime }(t),u_{m}^{\prime \prime }(t)\right)}_{\Gamma_{N}}-\gamma {(g(t)\frac{d}{dt}\alpha_{\epsilon }([[u_{m\tau }^{\prime }(t)]]),[[u_{m\tau }^{\prime }(t)]])}_{\Gamma_{c}}-{(g^{\prime }(t),\frac{d}{dt}\varphi_{\epsilon }([[u_{m\tau }^{\prime }(t)]]))}_{\Gamma_{c}}. \end{array}$$

Integration of both sides with respect to t yields

$$\begin{array}{rl} & \frac{\rho }{2}\|u_{m}^{\prime \prime }(t){\|}_{H}^{2}+\frac{1}{2}\|u_{m}^{\prime }(t){\|}_{V}^{2}+\frac{\gamma }{2}\int_{0}^{t}\|u_{m}^{\prime }(s){\|}_{V}^{2}\;ds+\rho \gamma [(u_{m}^{\prime \prime }(s),u_{m}^{\prime }(s)){]}_{0}^{t}\\ & \;\;-\rho \gamma \int_{0}^{t}\|u_{m}^{\prime \prime }(s){\|}_{H}^{2}\;ds\\ \leq & \frac{\rho }{2}\|u_{m}^{\prime \prime }(0){\|}_{H}^{2}+\frac{1}{2}\|{\dot{u}}_{0}{\|}_{V}^{2}+C(\gamma +1)(\|f^{\prime }{\|}_{L^{2}(0,T;H)}^{2}+\|F^{\prime }{\|}_{L^{2}(0,T;L^{2}(\Gamma_{N}))}^{2}+\|g^{\prime }{\|}_{L^{2}(0,T;L^{2}(\Gamma_{c}))}^{2})\\ & \;\;+\int_{0}^{t}\rho \|u_{m}^{\prime \prime }(s){\|}_{H}^{2}\;ds+[{\left(F^{\prime }(s),u_{m}^{\prime }(s)\right)}_{\Gamma_{N}}{]}_{0}^{t}-\int_{0}^{t}{\left(F^{\prime \prime }(s),u_{m}^{\prime }(s)\right)}_{\Gamma_{N}}\;ds\\ & \;\;-\gamma [{(g(s)\alpha_{\epsilon }([[u_{m\tau }^{\prime }(s)]]),[[u_{m\tau }^{\prime }(s)]])}_{\Gamma_{c}}{]}_{0}^{t}+\gamma \int_{0}^{t}{(g^{\prime }(s)\alpha_{\epsilon }([[u_{m\tau }^{\prime }(s)]]),[[u_{m\tau }^{\prime }(s)]])}_{\Gamma_{c}}\;ds\\ & \;\;+\gamma \int_{0}^{t}(g(s),\underset{=(d/ds)\varphi_{\epsilon }([[u_{m\tau }^{\prime }(s)]])}{\underbrace{\alpha_{\epsilon }([[u_{m\tau }^{\prime }(s)]])[[u_{m\tau }^{\prime \prime }(s)]]}}{)}_{\Gamma_{c}}\;ds\\ & \;\;-[{(g^{\prime }(s),\varphi_{\epsilon }([[u_{m\tau }^{\prime }(s)]])}_{\Gamma_{c}}{]}_{0}^{t}+\int_{0}^{t}{(g^{\prime \prime }(s),\varphi_{\epsilon }([[u_{m\tau }^{\prime }(s)]])}_{\Gamma_{c}}\;ds, \end{array}$$

where the eighth term on the right-hand side equals

$$\gamma [{(g(s),\varphi_{\epsilon }([[u_{m\tau }^{\prime }(s)]]))}_{\Gamma_{c}}{]}_{0}^{t}-\gamma \int_{0}^{t}{(g^{\prime }(s),\varphi_{\epsilon }([[u_{m\tau }^{\prime }(s)]]))}_{\Gamma_{c}}\;ds.$$

Hölder’s and Young’s inequalities, combined with the relations

$$H^{1}(0,T;L^{2}(\Gamma_{c}))↪C([0,T];L^{2}(\Gamma_{c})),$$

$\|[[v]]{\|}_{L^{2}(\Gamma_{c})}\leq C\|v{\|}_{V}$ and with $|\alpha_{\epsilon }(\cdot )|\leq 1$, $\varphi_{\epsilon }(\cdot )=\sqrt{|\cdot {|}^{2}+\epsilon^{2}}$, lead to

4.6 $$\begin{array}{rl} & \rho \|u_{m}^{\prime \prime }(t){\|}_{H}^{2}+\frac{1}{2}\|u_{m}^{\prime }(t){\|}_{V}^{2}+\gamma \int_{0}^{t}\|u_{m}^{\prime }(s){\|}_{V}^{2}\;ds+\rho \gamma \frac{d}{dt}\|u_{m}^{\prime }(t){\|}_{H}^{2}\\ & \;\;\leq C(\gamma +1)(\|u_{m}^{\prime \prime }(0){\|}_{H}^{2}+\|f{\|}_{H^{1}(0,T;H)}^{2}+\|F{\|}_{H^{2}(0,T;L^{2}(\Gamma_{N}))}^{2}+\|g{\|}_{H^{2}(0,T;L^{2}(\Gamma_{c}))}^{2}+\|{\dot{u}}_{0}{\|}_{V}^{2}+\epsilon^{2})\\ & \;\;+C(\gamma +1)\int_{0}^{t}(\rho \|u_{m}^{\prime \prime }(s){\|}_{H}^{2}+\frac{1}{2}\|u_{m}^{\prime }(s){\|}_{V}^{2})\;ds+C\gamma^{2}\|g{\|}_{H^{1}(0,T;L^{2}(\Gamma_{c}))}^{2}\;\forall t\in (0,T), \end{array}$$

where the last contribution owes to $\gamma (g(t)\alpha_{\epsilon }{\left([[u_{m\tau }^{\prime }(t)]]),[[u_{m\tau }^{\prime }(t)]]\right)}_{\Gamma_{c}}$ and $\gamma (g(t),\varphi_{\epsilon }{\left([[u_{m\tau }^{\prime }(t)]])\right)}_{\Gamma_{c}}$.

It remains to estimate $\|u^{\prime \prime }(0){\|}_{H}$. For this purpose we make t=0 and take $v=u^{\prime \prime }(0)\in V_{m}$ in (4.1) to see

$$\begin{array}{rl} & \rho \|u_{m}^{\prime \prime }(0){\|}_{H}^{2}+a(u_{0},u_{m}^{\prime \prime }(0))+{(\beta_{\epsilon }([[\gamma u_{0\nu }+{\dot{u}}_{0\nu }]]),[[u_{m\nu }^{\prime \prime }(0)]])}_{\Gamma_{c}}+{(g(0)\alpha_{\epsilon }([[{\dot{u}}_{0\tau }]]),[[u_{m\tau }^{\prime \prime }(0)]])}_{\Gamma_{c}}\\ & \;\;=(\rho f(0),u_{m}^{\prime \prime }(0))+{\left(F(0),u_{m}^{\prime \prime }(0)\right)}_{\Gamma_{N}}. \end{array}$$

Noting that

$$\begin{array}{rl} & a(u_{0},u_{m}^{\prime \prime }(0))\\ & \;\;=(-\mathrm{div}\sigma (u_{0}),u_{m}^{\prime \prime }(0))+{(\sigma (u_{0})\nu ,u_{m}^{\prime \prime }(0))}_{\Gamma_{N}}-{\left(\sigma_{\nu }(u_{0}),[[u_{m\nu }^{\prime \prime }(0)]]\right)}_{\Gamma_{c}}-{\left(\sigma_{\tau }(u_{0}),[[u_{m\tau }^{\prime \prime }(0)]]\right)}_{\Gamma_{c}} \end{array}$$

and using the compatibility conditions, we deduce

$$\rho \|u_{m}^{\prime \prime }(0){\|}_{H}^{2}=(\mathrm{div}\sigma (u_{0})+\rho f(0),u_{m}^{\prime \prime }(0)),$$

which implies $\left\|u_{m}^{\prime \prime }(0){\|}_{H}\leq C(\|\mathrm{div}\sigma (u_{0}){\|}_{H}+\|f(0){\|}_{H}\right)$.

Substituting this into (4.6), we proceed as in the previous subsection assuming ϵ≤1. If γ=0, Gronwall’s inequality gives us

$$\rho \|u_{m}^{\prime \prime }(t){\|}_{H}^{2}+\frac{1}{2}\|u_{m}^{\prime }(t){\|}_{V}^{2}\leq C_{4}(f,F,g,u_{0},{\dot{u}}_{0})\;e^{Ct}.$$

If γ>0, we further integrate (4.6) to have

$$B_{2}(t)+\rho \gamma \|u_{m}^{\prime }(t){\|}_{H}^{2}\leq C_{5}(f,F,g,u_{0},{\dot{u}}_{0},T){\left(\gamma +1\right)}^{2}+C(\gamma +1)\int_{0}^{t}B_{2}(s)\;ds,$$

where $B_{2}(t):=\int_{0}^{t}(\rho \|u_{m}^{\prime \prime }(t){\|}_{H}^{2}+\frac{1}{2}\|u_{m}^{\prime }(t){\|}_{V}^{2})\;ds$. Applying Gronwall’s inequality above and substituting the resulting estimate into (4.6), in which $2\rho \gamma |(u_{m}^{\prime \prime }(t),u_{m}^{\prime }(t))|$ is bounded by $\frac{\rho }{2}\|u_{m}^{\prime \prime }(t){\|}_{H}^{2}+2\rho \gamma^{2}\|u_{m}^{\prime }(t){\|}_{H}^{2}$, we conclude

4.7 $$\rho \|u_{m}^{\prime \prime }(t){\|}_{H}^{2}+\|u_{m}^{\prime }(t){\|}_{V}^{2}\leq C_{6}(f,F,g,u_{0},{\dot{u}}_{0},T){\left(\gamma +1\right)}^{3}\;e^{C(\gamma +1)t}.$$

### (d) Passage to limit

The argument of the passage to the limits m→∞ and ϵ→0 is basically similar to ([15], Section 3.7), the essential difference lying in the verification of the constraint $\gamma u(t)+u^{\prime }(t)\in K$. However, for the sake of completeness we present the whole proof.

First let us consider the limit m→∞ for fixed ϵ∈(0,1]. As a consequence of the *a priori* estimates (4.4) and (4.7), there exist a subsequence of $\left\{u_{m}\right\}$, denoted by the same symbol, and some $u_{\epsilon }\in W^{2,\infty }(0,T;H)\cap W^{1,\infty }(0,T;V)$ such that

$$\begin{array}{rl} & u_{m}⇀u_{\epsilon }\;\text{weakly-* in}\;L^{\infty }(0,T;V),\\ & u_{m}^{\prime }⇀u_{\epsilon }^{\prime }\;\text{weakly-* in}\;L^{\infty }(0,T;V)\\ \mathrm{and}\; & u_{m}^{\prime \prime }⇀u_{\epsilon }^{\prime \prime }\;\text{weakly-* in}\;L^{\infty }(0,T;H), \end{array}$$

as m→∞. Here, we note the compact embedding $W^{1,\infty }(0,T;L^{2}(\Omega_{\pm }))\cap L^{\infty }(0,T;H^{1}(\Omega_{\pm }))↪C([0,T];L^{2}(\Omega_{\pm }))$ (see [19]) and the compactness of the trace operator $H^{1}(\Omega_{\pm })\to L^{3}(\Gamma_{c})$ (e.g. [20]). It then follows that

4.8 $$\begin{array}{rl} & u_{m}\to u_{\epsilon }\;\text{and}\;u_{m}^{\prime }\to u_{\epsilon }^{\prime }\;\text{strongly in}\;C([0,T];H),\\ & \;[[u_{m}]]\to [[u_{\epsilon }]]\;\text{and}\;[[u_{m}^{\prime }]]\to [[u_{\epsilon }^{\prime }]]\;\text{strongly in}\;C([0,T];L^{3}(\Gamma_{c})), \end{array}$$

as m→∞. In particular, the initial conditions $u_{\epsilon }(0)=u_{0}$ and $u_{\epsilon }^{\prime }(0)={\dot{u}}_{0}$ hold. By choosing a further subsequence, we may also assume that

$$[[u_{m}]]\to [[u_{\epsilon }]]\;\text{and}\;[[u_{m}^{\prime }]]\to [[u_{\epsilon }^{\prime }]]\;\text{a.e.\textbackslash{} in}\;(0,T)\times \Gamma_{c}.$$

For arbitrary $\eta \in C_{0}^{\infty }(0,T)$ and $v\in V_{m}\;(m=2,3,\ldots )$, we find from (4.1) that

$$\begin{array}{rl} & \;\int_{0}^{T}\eta (t)(\rho (u_{m}^{\prime \prime }(t),v)+a(u_{m}(t),v)+{(\beta_{\epsilon }([[\gamma u_{m\nu }(t)+u_{m\nu }^{\prime }(t)]]),[[v_{\nu }]])}_{\Gamma_{c}}\\ & \;\;+{(g(t)\alpha_{\epsilon }([[u_{m\tau }^{\prime }(t)]]),[[v_{\tau }]])}_{\Gamma_{c}}-\rho (f(t),v)-{\left(F(t),v\right)}_{\Gamma_{N}})\;dt=0. \end{array}$$

Letting m→∞, using (4.8), and applying the dominated convergence theorem, we have

$$\begin{array}{rl} & \;\int_{0}^{T}\eta (t)(\rho (u_{\epsilon }^{\prime \prime }(t),v)+a(u_{\epsilon }(t),v)+{(\beta_{\epsilon }([[\gamma u_{\epsilon \nu }(t)+u_{\epsilon \nu }^{\prime }(t)]]),[[v_{\nu }]])}_{\Gamma_{c}}\\ & \;\;+{(g(t)\alpha_{\epsilon }([[u_{\epsilon \tau }^{\prime }(t)]]),[[v_{\tau }]])}_{\Gamma_{c}}-\rho (f(t),v)-{\left(F(t),v\right)}_{\Gamma_{N}})\;dt=0. \end{array}$$

Since $\bar{\overset{\infty }{\underset{m=1}{⋃}}V_{m}}=V$ and η is arbitrary, we conclude (3.7), that is, $u_{\epsilon }$ is a solution of **(VE)${\;}_{\epsilon }$** and also of **(VI)${\;}_{\epsilon }$** by virtue of proposition 3.6. Moreover, by making m→∞ in (4.4), (4.5) and (4.7), we also obtain

4.9 $$\begin{array}{rl} & \;\|u_{\epsilon }{\|}_{W^{2,\infty }(0,T;H)}^{2}+\|u_{\epsilon }{\|}_{W^{1,\infty }(0,T;V)}^{2}+\frac{1}{\epsilon }\int_{0}^{T}\|{\left[[\gamma u_{\epsilon \nu }(s)+u_{\epsilon \nu }^{\prime }(s)]\right]}_{-}{\|}_{L^{3}(\Gamma_{c})}^{3}\;ds\\ & \;\;\leq C(f,F,g,u_{0},{\dot{u}}_{0},T,\gamma ). \end{array}$$

Next we consider the limit ϵ→0. By (4.9), there exist a subsequence of $\left\{u_{\epsilon }\right\}$, denoted by the same symbol, and some $u\in W^{2,\infty }(0,T;H)\cap W^{1,\infty }(0,T;V)$ such that

$$\begin{array}{rl} & u_{\epsilon }⇀u\;\text{weakly-* in}\;L^{\infty }(0,T;V),\\ & u_{\epsilon }^{\prime }⇀u^{\prime }\;\text{weakly-* in}\;L^{\infty }(0,T;V)\\ \mathrm{and}\; & u_{\epsilon }^{\prime \prime }⇀u^{\prime \prime }\;\text{weakly-* in}\;L^{\infty }(0,T;H), \end{array}$$

as ϵ→∞. We observe from the third term on the left-hand side of (4.9) that

$$\int_{0}^{T}\|{\left[[\gamma u_{n}(t)+u_{n}^{\prime }(t)]\right]}_{-}{\|}_{L^{3}(\Gamma_{c})}^{3}\;dt=\lim_{\epsilon \to 0}\int_{0}^{T}\|{\left[[\gamma u_{\epsilon \nu }(t)+u_{\epsilon \nu }^{\prime }(t)]\right]}_{-}{\|}_{L^{3}(\Gamma_{c})}^{3}\;dt=0,$$

which verifies $[[\gamma u_{n}(t)+u_{n}^{\prime }(t)]]\geq 0$ a.e. on $(0,T)\times \Gamma_{c}$, that is, $\gamma u(t)+u^{\prime }(t)\in K$ for t∈(0,T).

For arbitrary $\tilde{v}\in L^{2}(0,T;K)$ we find from (3.6) that

$$\begin{array}{rl} & \;\int_{0}^{T}(\rho (u_{\epsilon }^{\prime \prime },\tilde{v}-(\gamma u_{\epsilon }+u_{\epsilon }^{\prime }))+a(u_{\epsilon },\tilde{v}-(\gamma u_{\epsilon }+u_{\epsilon }^{\prime }))+{(g,\varphi_{\epsilon }([[{\tilde{v}}_{\tau }-\gamma u_{\epsilon \tau }]])-\varphi_{\epsilon }([[u_{\epsilon \tau }^{\prime }]]))}_{\Gamma_{c}}\\ & \;\;-\rho (f,\tilde{v}-(\gamma u_{\epsilon }+u_{\epsilon }^{\prime }))-{\left(F,\tilde{v}-(\gamma u_{\epsilon }+u_{\epsilon }^{\prime })\right)}_{\Gamma_{N}})\;dt\geq 0, \end{array}$$

because $\psi_{\epsilon }([[{\tilde{v}}_{\nu }(t)]])=0$ and $\psi_{\epsilon }([[\gamma u_{\epsilon \nu }(t)+u_{\epsilon \nu }^{\prime }(t)]])\geq 0$. Consequently,

4.10 $$\begin{array}{rl} & \;\int_{0}^{T}(\rho (u_{\epsilon }^{\prime \prime },\tilde{v}-\gamma u_{\epsilon })+a(u_{\epsilon },\tilde{v})+{(g,\varphi_{\epsilon }([[{\tilde{v}}_{\tau }-\gamma u_{\epsilon \tau }]])-\varphi_{\epsilon }([[u_{\epsilon \tau }^{\prime }]]))}_{\Gamma_{c}}\\ & \;\;-\rho (f,\tilde{v}-(\gamma u_{\epsilon }+u_{\epsilon }^{\prime }))-{\left(F,\tilde{v}-(\gamma u_{\epsilon }+u_{\epsilon }^{\prime })\right)}_{\Gamma_{N}})\;dt\\ & \;\geq \int_{0}^{T}(\rho (u_{\epsilon }^{\prime \prime },u_{\epsilon }^{\prime })+a(u_{\epsilon },u_{\epsilon }^{\prime })+\gamma a(u_{\epsilon },u_{\epsilon }))\;dt\\ & \;=\frac{1}{2}(\rho \|u_{\epsilon }^{\prime }(T){\|}_{H}^{2}+\|u_{\epsilon }(T){\|}_{V}^{2})-\frac{1}{2}(\rho \|{\dot{u}}_{0}{\|}_{H}^{2}+\|u_{0}{\|}_{V}^{2})+\gamma \|u_{\epsilon }{\|}_{L^{2}(0,T;V)}^{2}. \end{array}$$

Here, observe that $\lim_{\epsilon \to 0}\|u_{\epsilon }^{\prime }(T){\|}_{H}^{2}=\|u^{\prime }(T){\|}_{H}^{2}$ in view of the compact embedding

$$W^{1,\infty }(0,T;L^{2}(\Omega_{\pm }))\cap L^{\infty }(0,T;H^{1}(\Omega_{\pm }))↪C([0,T];L^{2}(\Omega_{\pm })).$$

We further find that $\varphi_{\epsilon }([[u_{\epsilon \tau }^{\prime }]])\to |[[u_{\tau }^{\prime }]]|$ in $C([0,T];L^{2}(\Gamma_{c}))$ as ϵ→0, and that

$$\|u(T){\|}_{V}^{2}\leq \underset{\epsilon \to 0}{lim inf}\|u_{\epsilon }(T){\|}_{V}^{2}\;\mathrm{and}\;\|u{\|}_{L^{2}(0,T;V)}^{2}\leq \underset{\epsilon \to 0}{lim inf}\|u_{\epsilon }{\|}_{L^{2}(0,T;V)}^{2}.$$

In fact, the former inequality above results from the following weak convergence:

$$\begin{array}{rl} a(u(T)-u_{\epsilon }(T),w) & \;=\int_{0}^{T}(a(u^{\prime }(t)-u_{\epsilon }^{\prime }(t),\eta (t)w)+a(u(t)-u_{\epsilon }(t),\eta^{\prime }(t)w))\;dt\\ & \;\to 0\;\forall w\in V,\;\epsilon \to 0, \end{array}$$

where $\eta \in C^{\infty }([0,\infty ])$ is chosen so that η(0)=0 and η(T)=1. Therefore, making ϵ→0 in (4.10) deduces

$$\begin{array}{rl} & \;\int_{0}^{T}(\rho (u^{\prime \prime },\tilde{v}-\gamma u)+a(u,\tilde{v})+{(g,|[[{\tilde{v}}_{\tau }-\gamma u_{\tau }]]|-|[[u_{\tau }^{\prime }]]|)}_{\Gamma_{c}}-\rho (f,\tilde{v}-(\gamma u+u^{\prime }))\\ & \;\;-{\left(F,\tilde{v}-(\gamma u+u^{\prime })\right)}_{\Gamma_{N}})\;dt\geq \int_{0}^{T}(\rho (u^{\prime \prime },u^{\prime })+a(u,u^{\prime })+\gamma a(u,u))\;dt, \end{array}$$

namely,

$$\begin{array}{rl} & \;\int_{0}^{T}(\rho (u^{\prime \prime },\tilde{v}-(\gamma u+u^{\prime }))+a(u,\tilde{v}-(\gamma u+u^{\prime }))+{(g,|[[{\tilde{v}}_{\tau }-\gamma u_{\tau }]]|-|[[u_{\tau }^{\prime }]]|)}_{\Gamma_{c}}\\ & \;\;-\rho (f,\tilde{v}-(\gamma u+u^{\prime }))-{\left(F,\tilde{v}-(\gamma u+u^{\prime })\right)}_{\Gamma_{N}})\;dt\geq 0. \end{array}$$

This implies the pointwise (in time) variational inequality (3.4) by a technique based on the Lebesgue differentiation theorem (see [14, pp. 57–58]). Thus the existence part of theorem 3.4 has been established.

### (e) Uniqueness

Before proceeding to the proof of the uniqueness part of theorem 3.4, we present some preparatory results.

**Lemma 4.2.**

*There exists a vector function* $N\in H^{1}(\Omega )$ *such that its trace satisfies*

$$N=\nu \;\text{on}\;\Gamma_{c},\;N=0\;\text{on}\;\partial \Omega .$$

**Proof.**

Let ${\tilde{\Gamma }}_{c}$ be a neighbourhood of ${\bar{\Gamma }}_{c}$ such that $\Gamma_{c}⋐{\tilde{\Gamma }}_{c}⋐\Gamma$. Then there exists $\tilde{\nu }\in H_{00}^{1/2}(\Gamma )$ such that $\tilde{\nu }=\nu$ on $\Gamma_{c}$ and $\tilde{\nu }=0$ on $\Gamma \setminus {\tilde{\Gamma }}_{c}$. Then one can find some $N_{\pm }\in H^{1}(\Omega_{\pm })$ whose trace to $\partial \Omega_{\pm }$ equals the zero extension of $\tilde{\nu }$ to $\partial \Omega_{\pm }$. If we define $N=N^{+}$ in $\Omega_{+}$ and $N=N^{-}$ in $\Omega_{-}$, this is a desired function.

Using this lemma we introduce, for v∈V,

$$\bar{v}:=v-(v\cdot N)N.$$

Note that $\|\bar{v}{\|}_{H}\leq C\|v{\|}_{H}$, $\|\bar{v}{\|}_{V}\leq C\|v{\|}_{V}$, and that $[[{\bar{v}}_{\nu }]]=0$, $\left[[{\bar{v}}_{\tau }]]=[[v_{\tau }]\right]$, $[[{\left((v\cdot N)N\right)}_{\tau }]]=0$ on $\Gamma_{c}$.

For any solution u of (3.4), we see that $\sigma_{\nu }(u)\in L^{2}(0,T;H_{00}^{1/2}{\left(\Gamma_{c}\right)}^{*})$ and $\sigma_{\tau }(u)\in L^{2}(0,T;H_{00}^{1/2}{\left(\Gamma_{c}\right)}^{*})$ are characterized by

$$\begin{array}{rl} & {\left\langle \sigma_{\nu }(u(t)),[[v_{\nu }]]\right\rangle }_{\Gamma_{c}}=-\rho (u^{\prime \prime }(t),v)-a(u(t),v)+\rho (f(t),v)+{\left(F(t),v\right)}_{\Gamma_{N}}\\ & \;\forall v\in V,\;[[v_{\tau }]]=0\;\text{on}\;\Gamma_{c} \end{array}$$

and

$$\begin{array}{rl} & {\left\langle \sigma_{\tau }(u(t)),[[v_{\tau }]]\right\rangle }_{\Gamma_{c}}=-\rho (u^{\prime \prime }(t),v)-a(u(t),v)+\rho (f(t),v)+{\left(F(t),v\right)}_{\Gamma_{N}}\\ & \;\forall v\in V,\;[[v_{\nu }]]=0\;\text{on}\;\Gamma_{c}, \end{array}$$

respectively. The next lemma is essentially a consequence of the monotonicity of β and α appearing in (3.5).

**Lemma 4.3.**

*If* $u_{1}$, $u_{2}$ *are two solutions of* (3.4), *then for a.e.* t∈(0,T)

$${\left\langle \sigma_{\nu }(u_{1}(t))-\sigma_{\nu }(u_{2}(t)),[[\gamma u_{1\nu }(t)+u_{1\nu }^{\prime }(t)]]-[[\gamma u_{2\nu }(t)+u_{2\nu }^{\prime }(t)]]\right\rangle }_{\Gamma_{c}}\geq 0$$

*and*

$${\left\langle \sigma_{\tau }(u_{1}(t))-\sigma_{\tau }(u_{2}(t)),[[u_{1\tau }^{\prime }(t)]]-[[u_{2\tau }^{\prime }(t)]]\right\rangle }_{\Gamma_{c}}\geq 0.$$

**Proof.**

Arguing in the same way as in proposition 3.1, we get

$${\left\langle \sigma_{\nu }(u_{i}),[[v_{\nu }]]\right\rangle }_{\Gamma_{c}}\leq 0\;\forall v\in K\;\text{and}\;{\left\langle \sigma_{\nu }(u_{i}),[[\gamma u_{i\nu }+u_{i\nu }^{\prime }]]\right\rangle }_{\Gamma_{c}}=0,$$

for i=1,2. The first desired inequality follows from these and $\gamma u_{i}+u_{i}^{\prime }\in K$.

Again by the same way as in proposition 3.1, we have

$${\left\langle \sigma (u_{i}),[[v_{\tau }]]\right\rangle }_{\Gamma_{c}}\leq {\left(g(t),|[[v_{\tau }]]|\right)}_{\Gamma_{c}}\;\forall v\in V\;\text{and}\;{\left\langle \sigma_{\tau }(u_{i}),[[u_{i\tau }^{\prime }]]\right\rangle }_{\Gamma_{c}}={\left(g(t),|[[u_{i\tau }^{\prime }]]|\right)}_{\Gamma_{c}},$$

for i=1,2, which lead to the second desired inequality.

Now we prove the uniqueness of a solution of (3.4). Let $u_{1}$, $u_{2}$ be two solutions of (3.4) and set $w:=u_{1}-u_{2}$. Then it follows that

$$\rho (w^{\prime \prime }(t),v)+a(w(t),v)+{\left\langle \sigma_{\nu }(w(t)),[[v_{\nu }]]\right\rangle }_{\Gamma_{c}}+{\left\langle \sigma_{\tau }(w(t)),[[v_{\tau }]]\right\rangle }_{\Gamma_{c}}=0\;\forall v\in V,\;\text{a.e.}\;t\in (0,T).$$

Taking $v=\gamma w(t)+w^{\prime }(t)$ and using lemma 4.3, we deduce that

$$\begin{array}{rl} & \frac{1}{2}\frac{d}{dt}(\rho \|w^{\prime }(t){\|}_{H}^{2}+\|w(t){\|}_{V}^{2})+\rho \gamma (w^{\prime \prime }(t),w(t))+\gamma \|w(t){\|}_{V}^{2}\\ & \;\;\leq -\gamma {\left\langle \sigma_{\tau }(w(t)),[[w_{\tau }(t)]]\right\rangle }_{\Gamma_{c}}\\ & \;\;=-\gamma {\left\langle \sigma_{\tau }(w(t)),[[{\bar{w}}_{\tau }(t)]]\right\rangle }_{\Gamma_{c}}\\ & \;\;=\rho \gamma (w^{\prime \prime }(t),\bar{w}(t))+\gamma a(w(t),\bar{w}(t)), \end{array}$$

which, combined with $\left(w^{\prime \prime }(t),w(t)-\bar{w}(t))=(w^{\prime \prime }(t)\cdot N,w(t)\cdot N\right)$, gives

$$\frac{1}{2}\frac{d}{dt}(\rho \|w^{\prime }(t){\|}_{H}^{2}+\|w(t){\|}_{V}^{2})+\rho \gamma (w^{\prime \prime }(t)\cdot N,w(t)\cdot N)\leq \gamma a(w(t),\bar{w}(t))\leq C\gamma \|w(t){\|}_{V}^{2}.$$

Integrate this with respect to t to obtain (note that $w(0)=w^{\prime }(0)=0$)

$$\begin{array}{rl} & \frac{1}{2}(\rho \|w^{\prime }(t){\|}_{H}^{2}+\|w(t){\|}_{V}^{2})+\frac{\rho \gamma }{2}\frac{d}{dt}\|w(t)\cdot N{\|}_{H}^{2}\leq \gamma \int_{0}^{t}(\rho \|w^{\prime }(s)\cdot N{\|}_{H}^{2}+C\|w(s){\|}_{V}^{2})\;ds\\ & \;\;\leq C\gamma \int_{0}^{t}(\rho \|w^{\prime }(s){\|}_{H}^{2}+\|w(s){\|}_{V}^{2})\;ds. \end{array}$$

Setting $D(t):=\int_{0}^{t}(\rho \|w^{\prime }(s){\|}_{H}^{2}+\|w(s){\|}_{V}^{2})\;ds$, we find from further integration of this estimate that

$$D(t)+\rho \gamma \|w(t)\cdot N{\|}_{H}^{2}\leq C\gamma \int_{0}^{t}D(s)\;ds.$$

By Gronwall’s inequality, D(t)≡0 and hence w(t)≡0, which shows the uniqueness.

The proof of theorem 3.4 has been completed.

## Data Availability

No new data were created or analysed in this study.

## References

[1] Broberg KB. 1999 Cracks and fracture. London, UK: Academic Press.

[2] Freund LB. 1990 Dynamic fracture mechanics. Cambridge, UK: Cambridge University Press.

[3] Udías A, Madariaga R, Buforn E. 2014 Source mechanisms of earthquakes: theory and practice. Cambridge, UK: Cambridge University Press.

[4] Itou H, Kovtunenko VA, Tani A. 2011 The interface crack with coulomb friction between two bonded dissimilar elastic media. Appl. Math. **56**, 69-97. (10.1007/s10492-011-0010-7)

[5] Khludnev AM, Kovtunenko VA. 2000 Analysis of cracks in solids. Southampton, UK: WIT Press.

[6] Andersson J. 2016 Optimal regularity for the Signorini problem and its free boundary. Invent. Math. **204**, 1-82. (10.1007/s00222-015-0608-6)

[7] Andersson J, Mikayelyan H. 2011 *C*^1, α^-regularity for solutions to the *p*-harmonic thin obstacle problem. *Int. Math. Res. Not. IMRN* **1**, 119-134. (10.1093/imrn/rnq061)

[8] Ovcharova N, Gwinner J. 2014 A study of regularization techniques of nondifferentiable optimization in view of application to hemivariational inequalities. J. Optim. Theory Appl. **162**, 754-778. (10.1007/s10957-014-0521-y)

[9] Lebeu G, Schatzman M. 1984 A wave problem in a half-space with a unilateral constraint at the boundary. J. Differ. Equ. **53**, 309-361. (10.1016/0022-0396(84)90030-5)

[10] Kim JU. 1989 A boundary thin obstacle problem for a wave equation. Commun. Partial. Differ. Equ. **14**, 1011-1026. (10.1080/03605308908820640)

[11] Eck C, Jarušek J, Krbec M. 2005 Unilateral contact problems: variational methods and existence theorems. Boca Raton, FL: CRC Press.

[12] Tani A. 2020 Dynamic unilateral contact problem with averaged friction for a viscoelastic body with cracks. In *Mathematical Analysis of Continuum Mechanics and Industrial Applications III - Proceedings of the International Conference CoMFoS18* (eds H Itou, S Hirano, M Kimura, VA Kovtunenko, AM Khludnev). Mathematics for Industry **34**, 3–21. Berlin, Germany: Springer.

[13] Petrov A, Schatzman M. 2009 Mathematical results on existence for viscoelasto dynamic problems with unilateral constraints. SIAM J. Math. Anal. **40**, 1882-1904. (10.1137/070695101)

[14] Duvaut G, Lions JL. 1976 Inequalities in mechanics and physics. Berlin, Germany: Springer.

[15] Itou H, Kashiwabara T. 2021 Unique solvability of crack problem with time-dependent friction condition in linearized elastodynamic body. Math. Notes NEFU **28**, 121-134. (10.25587/SVFU.2021.38.33.008) PMC9510035 36154480

[16] Cocou M, Scarella G. 2006 Analysis of a dynamic unilateral contact problem for a cracked viscoelastic body. Z. Angew. Math. Phys. **57**, 523-546. (10.1007/s00033-005-0013-x)

[17] Kikuuwe R, Brogliato B. 2017 A new representation of systems with frictional unilateral constraints and its Baumgarte-like relaxation. Multibody Syst. Dyn. **39**, 267-290. (10.1007/s11044-015-9491-6)

[18] Lions JL, Magenes E. 1972 Non-homogeneous boundary value problems and applications. Berlin, Heidelberg, Germany: Springer.

[19] Simon J. 1986 Compact sets in the space $L^{p}(0,T;B)$. Ann. Mat. Pura Appl. **146**, 65-96. (10.1007/BF01762360)

[20] Nečas J. 2012 Direct methods in the theory of elliptic equations. Berlin, Heidelberg, Germany: Springer.

